# Books and the War

**Published:** 1919-08-02

**Authors:** 


### "BOOKS AND THE WAR."

### By "BLUE TABS."

"Dreams, books, are each a world; and books, we know,
Are a substantial world. . . "

CHARLES LAMB has written of the "fearful joy" of stolen reading—snatched from the books displayed at a street stall; but his sympathetic soul would have appreciated still more the delight of reading on active service. Only those who have experienced it can realise the relief and real relaxation of withdrawing oneself from the horrors and sordid, dreary details of modern warfare into the realm of romance and imagination. Fancy the delight of turning from the sharp anxiety, or the dull monotony, of life at the Front to the mingled humour and thrills of *Lorna Doone*, or the polished villainies of John Silver, or the quaint "observations" of Captain Cuttle and Sam Weller. During a "tour" in the trenches there was little chance or inclination for reading—eating and sleeping absorbed the daylight hours—what reading there was consisted of the scanning of some newspaper—generally an old one—but more from a sense of boredom than with any idea of enjoyment. But when the time had come to be relieved and you were back in "rest billets," then out would come some well-worn little volume and for a time even the ever-present rumble of guns would fade into oblivion. There were times, too, when a need was felt for lighter stuff, when a Red Cross parcel of magazines was ransacked and the latest *Pearson's* or *Strand* was all that one desired—but this could not be described as "reading" in the true, or *Elian* sense. A time when such artificial pleasures were most acceptable was when on night duty in an office near the Front. The constant telephone calls, the irritating interruptions of every kind, made one little inclined for more substantial fare; short stories are always more adapted to desultory reading, and their quickly developed situations seemed very appropriate to the brief respites from work. One curious feature of the world's—the outside, far-away, world's—doings, as related in the newspapers, was that they seemed so unreal; the only real things

**'Books and the War"**—(*continued*).

seemed to be those of one's daily life, everything else was seen through a cloud of memory—like reflections in a hazy mirror.

WHAT THE SOLDIER READS.

It is obviously impossible to dogmatise on the tastes of such an Army as that which was recently in being—drawn, as it was, from every class of society and from so many and such varied countries—but, all the same one author's books were more universally asked for than any other's. To many of us the sporting novels of the late Nat Gould represent a phase of our literary progress that was left behind a good many years ago, but that he is an abiding favourite, or perhaps one should say, an acquired taste, with a very large number of those who composed our citizen army is evident from the continuous demand for his books in the hospitals and rest stations in all theatres of war. Among other popular writers were Hall Caine, Marie Corelli, Harrison Ainsworth, Charles Garvice, Jack London, Conan Doyle, and Barry Pain. At officers' hospitals the books of W. W. Jacobs, Anthony Hope, John Buchan, H. G. Wells, and Rider Haggard were also asked for. The subaltern of the New Army does not apparently read Dickens, Thackeray, or Scott, and even the more recent works of Seton Merriman, which were literally devoured as soon as they were published, seem to have been forgotten, for the time being at any rate. Books on boxing were popular with all ranks. The books written concerning the different phases of warfare seemed more appreciated by people at home than by those whose lives they depicted with more or less accuracy.

The *Daily Mail* was as ubiquitous in France as it is in England, and the daily illustrated papers—the *Daily Mirror* and *Daily Sketch*—were bought up as soon as they were available for sale. *John Bull* could be seen in every kind of place, and was always widely read. In the earlier days of the War *Land and Water* had a great vogue; but this died out latterly, though *Truth* took the place of the former favourite to a certain extent. Of the other weekly papers *Answers* and *Tit-Bits* were always welcomed in the hospitals, but, as with the "dailies," an illustrated paper, such as the *Sphere*, was always chosen in preference to any other. *Punch* was not often seen and did not appeal to such a large audience as *Blighty*. The only serious monthly journal that one ever came across on active service was *Blackwood's*.

A MEDICAL OFFICER'S LIBRARY.

The number of books that could be taken on active service was naturally very limited; kits were kept rigidly down to the authorised weight, and, where it was a choice between boots, or spare underclothes, and books, the latter had to be left out—no matter how reluctant one might be to do so. But, of course, there was no restriction on what one could have sent out afterwards, and in this way many men accumulated quite a little library by the time it was necessary to think of demobilisation and the return home. Personally, I found it necessary to have quite a lot of books of reference in connection with my special work—hygiene—but a regimental medical officer would not require many technical books, and, as he was constantly on the move and always had to travel "light," he usually preferred to be without them. In my own case I sent home an old German medicine-chest nearly filled with books when I left France for good, but the problem of carrying them round with me was not easy to solve on several occasions. During the retreat in March of last year all my books and much "surplus" kit of various kinds went down to the Base to wait for happier times and a more abiding resting-place. When I went out to France I took one little volume only—apart from technical books—and that was "Treasure Island" and "Kidnapped," in a cheap edition under one cover: it is a couple that I find hard to beat where the choice is limited, and many a refreshing half-hour did I spend in this good company. Another volume that became a great favourite with me, and was read again and again, was "The Thirty-nine Steps" and "The Power House"—in a similar form. If a third and fourth had been possible they would probably have been "Many Cargoes," "Kipps," and one of Kipling's books of short stories—preferably, "The Jungle Book."

It was not until some time after the Armistice that one could settle down to a good feast of literary fare: fortunately, by that time, camp libraries had been established in many places, and it was possible to get hold of many old favourites and to find new friends. Here is a list of some that were read between November and April: Collections of short stories by Poe, Hawthorne, Bret Harte, Balzac, Merimée, Hilaire Belloc, H. G. Wells, Maupassant and Daudet, Stevenson's "Art of Writing," and "Inland Voyage." The latter was particularly interesting, in that I was at the time in the very district described by the author in very different circumstances. My previous admiration for Joseph Conrad was increased by dipping further into his writings, including "Typhoon," "Youth," "Almayer's Folly," and "Lord Jim"—for the third time! A book of Tolstoi's tales and "The Club of Queer Trades," by G. K. Chesterton, came next, and one more, one of my greatest favourites, "The Crock of Gold." If ever we have another war—*absit omen!*—and I go a-warring again my original choice of the Stevenson pair would still be my first choice, with the last-named gem of Irish humour as a second string; these are books that would have come into Ruskin's second division—not "books of the hour," but "books of all time." In conclusion, one may add, as Isaac Barrow wrote, that on active service—as elsewhere—"He that loveth a book will never want a faithful friend, a wholesome counsellor, a cheerful companion, an effectual comforter."

